# Association of frailty with functional difficulty in older Ghanaians: stability between women and men in two samples with different income levels

**DOI:** 10.1186/s12877-024-05534-9

**Published:** 2024-11-15

**Authors:** Nestor Asiamah, Emelia Danquah, Edgar Ramos Vieira, Peter Hjorth, Reginald Arthur-Mensah Jnr, Simon Mawulorm Agyemang, Hafiz T. A. Khan, Cosmos Yarfi, Faith Muhonja

**Affiliations:** 1 Division of Interdisciplinary Research and Practice, School of Health and Social Care, Colchester , Essex, CO4 3SQ UK; 2 International Public Health Management Programme, University of Europe for Applied Sciences, Iserlohn, Reiterweg 26B, 58636 Germany; 3 Research Faculty, Berlin, School of Business and Innovation , 97-99 Karl Marx Strasse. 12043, Berlin, Germany; 4 Department of Gerontology and Geriatrics, Africa Centre for Epidemiology, P. O. Box AN, Accra North, Accra, 18462 Ghana; 5 Research Directorate, Koforidua Technical University, Koforidua, E/R Ghana; 6 Department of Physical Therapy, Florida International UniversityNicole Wertheim College of Nursing & Health SciencesInternational University, Florida, USA; 7 Institute of Regional Health Research, University of Southern Denmark, Odense, 5000 Denmark; 8 Department of Nursing and Midwifery, Faculty of Health and Allied Sciences, Pentecost University, AccraAccra, P.O. Box KN 1739, Ghana; 9 Department of Science/Health, Physical Education and Sports, E/R, Abetifi Presbyterian College of Education, Abetifi, P.O. Box 19, Abetifi , Ghana; 10 College of Nursing, Midwifery, and Healthcare, University of West London, Paragon House, Boston Manor Road, Brentford, TW8 9GB UK; 11 Department of Physiotherapy and Rehabilitation Sciences, University of Health and Allied Sciences, PMB 31, Ho, Ghana; 12 School of Public Health, Department of Community Health, Amref International University, P. O. Box27691 – 00506, , Nairobi, Kenya

**Keywords:** Frailty, Functional difficulty, Older adults, Gender, Income, Ghana

## Abstract

**Background:**

Research to date suggests that frailty is higher in women and is associated with functional difficulty. This study builds on the evidence by examining the association between frailty and functional difficulty between low- and higher-income groups and between older men and women in these income groups.

**Methods:**

This study adopted a cross-sectional design that complied with the STROBE checklist and included steps against confounding and common methods bias. The population was community-dwelling older adults aged 50 years or older in two urban neighbourhoods in Accra, Ghana. Participants were either in the low-income group in a low socioeconomic neighbourhood (*n* = 704) or the higher-income group in a high socioeconomic neighbourhood (*n* = 510). The minimum sample necessary was calculated, and the hierarchical linear regression analysis was utilised to analyse the data.

**Results:**

Frailty was positively associated with functional difficulty in the low- and higher-income samples, but this association was stronger in the higher-income sample. Frailty was positively associated with frailty in men and women within the low- and higher-income samples.

**Conclusion:**

The association of frailty with functional difficulty was consistent between low- and higher-income samples, although the strength of the relationship differed between these samples. In both income samples, the foregoing relationship was consistent between men and women, although the strength of the relationship differed between men and women.

**Supplementary Information:**

The online version contains supplementary material available at 10.1186/s12877-024-05534-9.

## Introduction

The world’s population is ageing more rapidly than ever due to increasing life expectancy and declining fertility [1, 2]. Consequently, the global population will become superaged by 2050 [2, 3]. A superaged population has at least 20% of its members being aged 65 years or older. Ghana and other West African countries have a relatively young population, so they are unlikely to become superaged by 2050, but the proportions of older adults aged 65 years or higher in Ghana are expected to increase significantly [4]. The ageing process accompanies physiological changes such as a loss of bone and muscle mass [5], and frailty may be an outcome of these changes [6, 7]. People with frailty are less capable to perform Activities of Daily Living (ADLs), which refer to daily self-care activities such as bathing, toileting, and dressing necessary for a normal life [8]. ADLs are necessary for happiness and quality of life [9]. Hence, interventions enabling ageing people to avoid the early onset of frailty are necessary. We define frailty as the limitations and impairments in physical performance including Instrumental Activities of Daily Living (IADLs) [8, 10]. Though several definitions of frailty exist, we chose this definition as it is suited for our measurement of frailty in the context of geriatric medicine [8]. Measuring frailty and functional difficulty in this context enabled us to reach evidence with clinical implications.

Functional difficulty is the extent to which people find it difficult to complete or perform self-care activities including ADLs and IADLs (e.g., walking to a nearby supermarket) [8]. The maintenance of ADLs and IADLs is a core indicator of healthy ageing, but this can dwindle as frailty increases. This thought is supported by studies [6, 11, 12] confirming that functional difficulty, which is analogous to functional limitation [8, 13], is higher at higher frailty. Thus, frailty can be positively associated with functional difficulty.

Over the past decade, significant research has been focused on how frailty and functional difficulty are influenced by socioeconomic factors. In their study, Gomes and colleagues [14] found that gender, age, and other personal characteristics were associated with worse frailty status among a sample of older adults from North America, South America, and Europe. Cross-sectional studies [15, 16] based on African samples have reported an association between higher frailty and poor self-reported health or chronic disease status. These studies suggest that people with at least one chronic disease or who perceived their health to be poor reported higher frailty levels. Many studies [17–21], have found that frailty is higher in women. In the study of Gomes [14], insufficient income was found to predict frailty. Thus, significant empirical evidence exists on the association of frailty with gender, income, and other indicators of socio-economic status.

On the other hand, research [22, 23] has confirmed an association between functional difficulty and demographic factors such as age and sex. Onadja and colleagues [22] found that functional difficulty was higher at older ages in Burkina Faso whereas Miszkurka [23] reported higher functional difficulty for women in a sample from Mali, Senegal, and Burkina Faso. Research has also shown that frailty is positively associated with functional difficulty, but limited research has examined this relationship [8, 13, 16] and no study in Africa has examined this association. The primary aim of this study, therefore, was to assess the association between frailty and functional difficulty based on an African sample.

The literature review above suggests that women and individuals with insufficient or lower income would report higher frailty. In Africa, women generally earn less income and may depend on the income of their spouses [24]. As such, they may have less access to social services [e.g., healthcare and exercise services] necessary for maintaining functional ability over the life course. This reasoning holds more meaning in an African context such as Ghana where most women are unemployed or live on lower income [4, 24]. This phenomenon may explain the association of frailty with functional difficulty and its stability between socioeconomic factors (e.g., gender and income level) in an African sample.

To build on the evidence to date, this study examined the association of frailty with functional difficulty and ascertained the consistency of this relationship between men and women and between groups with different income levels. This analysis is necessary because the evidence to date is limited to the frailty-functional-difficulty nexus and the relationship of frailty and functional difficulty with socioeconomic factors. There has been no evaluation of the stability of the association between frailty and functional difficulty between personal variables such as income and gender. Evidence about whether the foregoing relationship is stable across subgroups of the population would improve stakeholders’ understanding of frailty as a health risk. It might inform clinicians about groups more likely to lose functional ability due to frailty and provide a basis for planning against the ageing population.

Older age, chronic disease status (CDS), and living alone or not being married are amongst the most frequently reported predictors of frailty [18–21, 25], which means that these personal factors can significantly influence the association of frailty with functional difficulty. Previous studies, however, have not systematically adjusted for these factors. Systematically controlling for these factors can consolidate the evidence to date and provide theoretical and practical implications. Given these concerns, this study aimed to answer the following research questions: (1) is there an association between frailty and functional difficulty; (2) is the association between frailty and functional difficulty consistent between older adults on low and higher incomes, and (3) is the association between frailty and functional difficulty stable between men and women in the low and higher-income groups? The implications of our findings for ageing, healthcare planning, and health behaviour are discussed.

## Methods

### Design

A cross-sectional design that follows the STROBE (Strengthening the Reporting of Observational Studies in Epidemiology) was adopted. This design included a Hierarchical Linear Regression (HLR) analysis and Common Methods Bias (CMB) assessment.

### Samples

The study participants were community-dwelling older adults aged 50 years or older in two neighbourhoods in Accra. The first neighbourhood was a low socioeconomic area mostly characterised by petty traders and workers with low income. The other neighbourhood was a higher socioeconomic area with residents who were mostly high-income earners (e.g., business executives, university academic staff and managers). The low socioeconomic area was the source of our low-income sample whereas the higher socioeconomic area was the source of our higher-income sample. We confirmed with an independent samples *t*-test that the higher-income sample (Mea*n* = ₵2,129 or 175 United States Dollars [USD]) had a mean income significantly higher than the mean income of the low-income sample (Mea*n* = ₵923 or 76 USD).

### Selection

Convenience sampling was employed to select individuals who were available for the study. The participants were selected with the following relevant inclusion criteria: (1) being aged 50 years or older; (2) having at least a basic school leaving certificate, which we used as an indicator of the ability to speak and write in English, the medium in which the survey was completed, and (3) availability and willingness to participate in the study. We administered the questionnaire in English because it was easier for the participants to write in this language, which was Ghana's official language. There was no sampling frame, so we recruited eligible individuals at community centres and social gatherings over four weeks with a structured interview lasting between 5–7 min. The number of older adults selected was 1484 (low income = 901; higher income = 583). We calculated the minimum sample size necessary with the G*Power software and recommended statistics (effect size = 0.2; α = 0.05; power = 0.8) [26]. The minimum sample necessary for HLR analysis with a maximum of 11 predictors was 95. We gathered data on all the eligible participants to maximise the power of our tests.

### Measures

Frailty and functional difficulty were measured with standard Likert-type scales. We measured these variables with standard scales developed for geriatric medical practice, enabling us to identify implications for clinical practice. Frailty was measured with the 15-item Chinese version of the Tilburg Frailty Indicator adopted in whole from Dong and colleagues[8] with its dichotomous descriptive anchors [i.e., no – 0; yes – 1]. This tool was used because it is relatively short and was, therefore, suited for older adults who may be unable to complete long surveys. A previous study has evidenced the scale’s reliability in a Ghanaian context [27].

Functional difficulty was measured with a 14-item tool with four descriptive anchors (i.e., no difficult – 1; somewhat difficult – 2; most difficult – 3 and could not perform – 4) adopted in whole from Nagarkar et al.[28]. This scale measures the extent to which a person found it difficult to perform self-care ADL and IADL over the past week. Both scales were internally consistent as they produced a Cronbach’s α coefficient ≥ 0.7 (Frailty = 0.79 and functional difficulty = 0.87), which made them transferable to the Ghanaian sample. We generated data on both scales by summing up their corresponding scores as recommended [8, 28, 29]. Appendices 1a and 1b respectively show items used to measure frailty and functional difficulty.

We measured personal variables and potential confounders (i.e., age, CDS, self-reported health, marital status, gender, income, and education) previously found to be associated with frailty and functional difficulty [20, 25]. Income was measured as a discrete variable by asking participants in the low ad higher income samples to report their gross monthly income in cedis. Age (in years) was a discrete variable measured by asking the participants to report their chronological age. Education was also a discrete variable measured as the individual’s number of years of schooling. The other confounders were measured as categorical variables in harmony with previous research [29, 30]. Thus, the participants were asked to report their self-reported health (poor – 1; good – 2), gender (male – 1; female – 2), CDS (none – 1; 1 or more – 2), and marital status (not married – 1; married – 2) by choosing a category that best described their situation. We coded categorical variables into dummy-type variables for HLR analyses. In dummy-coding of the variables, categories were coded into binary data (i.e., 0 and 1). In the regression models, one group within dummy-type variables was set as a reference.

### Instrumentation

The variables were measured with a self-reported questionnaire with three parts. The first part was a preamble introducing the study, ethical statement, and survey completion instructions. The second part presented measures of frailty and functional difficulty whereas the final section presented questions measuring the potential confounders and personal variables. We followed two steps previously used [30, 31] to avoid and assess CMB. The first step involved using standardised measures and structuring the questionnaire according to recommended practices. The second step involving factor analysis (based on the maximum likelihood method) and the Harman's one-factor approach evidenced the absence of CMB in the data.

### Ethics and data collection

The questionnaires were hand-delivered to the participants by trained research assistants. The participants returned completed questionnaires instantly or after two weeks, depending on what worked for them. Data were collected over four weeks (November 4 – December 5, 2022). We analysed 1214 questionnaires (the low-income group = 704; the high-income group = 510) out of 1227 returned after discarding 13 that were not completed at all or were completed halfway. The response rates of this study were 78% and 87% for the low- and higher-income groups respectively. The response rate for the low-income group was lower, although more individuals in this group were available to complete the questionnaire.

### The statistical strategy

We analysed the data with SPSS 28 [IBM SPSS Inc., New York] in two phases. In the initial exploratory phase, we summarised the data, assessed five statistical assumptions necessary for HLR analysis, and performed the first sensitivity analysis to identify the ultimate confounders. Continuous or discrete variables were summarised with the mean whereas categorical variables were summarised with frequencies. Following previous research [29], we analysed the data with their missing items since the missing data were associated with only two potential confounders and were randomly distributed. Missing data were the consequence of the participants choosing not to respond to a question or item. Appendix 2a shows the steps taken to assess the five assumptions for using HLR analysis. The first sensitivity analysis adopted from previous research [29, 30] aimed to identify the strongest confounders of the primary association (i.e., the association between frailty and functional difficulty) and to remove variables that would not confound this association. This approach enabled us to assess the relative influences of the confounders on the primary relationship. Appendix 2b shows the steps taken in this analysis. Age and CDS were the ultimate confounders found in this analysis and were incorporated into the final models, hereby called the ultimate models.

**Fig. 1:**
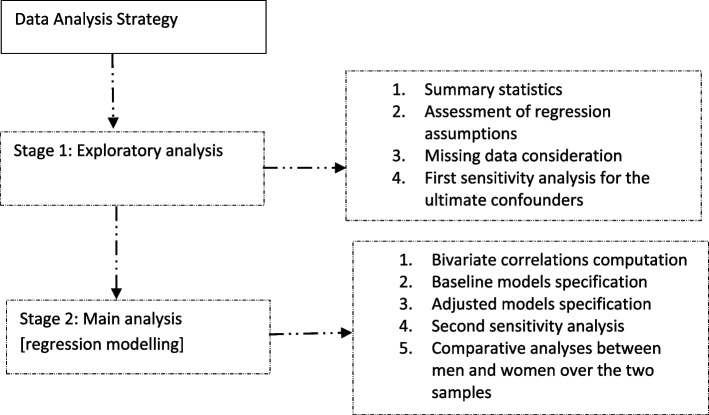
A flow chart of the statistical analysis strategy employed

In the second phase, we computed Pearson’s correlations for both samples as a basis for HLR analysis. To complement our analyses, we included Pearson’s correlation between gender and frailty. A baseline HLR model (model 1) was then fitted to assess the association between frailty and functional difficulty over both samples. We then built on model 1 by adjusting for age and CDS in models 2 and 3 respectively. The ultimate model (model 4) was subsequently fitted by adjusting for age and CDS together. We computed the percentage change in the standardised coefficient (β) between model 1 and each of the other models (models 2, 3 and 4), enabling us to see how much age and CD individually (in models 2 and 3) and jointly (in model 4) influenced the primary association over the two samples. The conclusions of this study were based on model 4, and the statistical significance of the result was detected at a minimum of *p* < 0.05. To complement results in the regression models, the independent samples t-test was used to stratify frailty and functional difficulty according to gender in the low and higher-income samples. Figure 1 is a flowchart of the statistical analysis strategy.

### Findings

Table 1 shows summary statistics on the two samples. About 52% (*n* = 368) of the low-income group and 48% (*n* = 245) of the high-income group were women. The average age of the low-income group was about 63 years (Mea*n* = 62.91; SD = 9.29) whereas the average age of the high-income group was about 59 years (Mea*n* = 57.87; SD = 8.75). Frailty in the low-income sample was higher (Mea*n* = 6.23; SD = 3.76). This outcome was possibly due to people in the low-income sample not having the financial resources to utilise health and social services that protect humans from frailty.

**Table 1:** Summary statistics of the two samples

Variable	Group	Sample 1 [Low income; *n* = 704]		Sample 2 [Higher income; *n* = 510]	
		n/Mean	%/SD	n/Mean	%/SD
Categorical variables					
Gender	Men	368	52.27	245	48.04
	Women	332	47.16	265	51.96
	Missing	4	0.57	0	0
Chronic disease status	None	187	26.56	330	64.71
	One or more	517	73.44	180	35.29
Self-reported health	Poor	191	27.13	135	26.47
	Good	513	72.87	375	73.53
Marital status	Not married	235	33.38	370	72.55
	Married	376	53.41	140	27.45
	Missing	93	13.21	0	0
	Total	704	100	510	100
Continuous/discrete variables					
Income [₵]	–-	922.77^a^	510.46	2129.41^a^	1583.96
Education [yrs]	–-	14.23	2.09	18.21	4.11
Age [yrs]	–-	62.91	9.29	57.87	8.75
Frailty	–-	6.23^b^	3.76	4.78^b^	2.77
Functional difficulty	–-	30.58	11.36	21.95	10.03

n – frequency; % – per cent; *SD *standard deviation; n and % apply to only categorical variables whereas the mean and SD apply to only continuous/discrete variables

^a^independent samples t-test showed a significant difference in income between samples 1 and 2 [t = -15.37, *p* < 0.001, two-tailed] based on “equal variances not assumed”

^b^independent samples t-test showed a significant difference in income between samples 1 and 2 [t = 7.75, p < 0.001, two-tailed] based on “equal variances not assumed”

Table 2 stratifies frailty and functional difficulty according to gender in the low and higher-income samples. In both samples, women reported higher frailty. Women reported higher functional difficulty only in the higher-income sample (t = -4.78; *p* < 0.001).

**Table 2:** Stratification of frailty and functional difficulty according to gender in the low- and higher-income samples

Variable	Group	Low income (*n* = 704)				High income (*n* = 510)			
		Mean	SD	t	p	Mean	SD	t	p
Frailty	Men	5.38	2.85	-3.78	***	6.01	2.74	-6.87	***
	Women	6.10	3.07			7.47	2.45		
Functional difficulty	Men	23.73	5.59	-1.56	0.060	20.79	7.65	-4.78	***
	Women	24.35	6.45			23.85	8.04		

^***^p < 0.001; the homogeneity of variances assumption is met at p > 0.05 for each independent samples t-test; SD – standard deviation (of the mean)

Table 3 shows relevant correlations between variables for the two samples. There was a positive correlation between frailty and functional difficulty in both the low-income group (r = 0.371; *p* < 0.001; two-tailed) and the high-income group (r = 0.535; *p* < 0.001; two-tailed), though this correlation was stronger in the high-income group.

**Table 3:** Bivariate correlations between frailty, functional difficulty, gender, and covariates

Variable	1	2	3	4	5
Sample 1 [Low income; *n* = 704]					
1. Frailty	1	.371**	.109**	.332**	.258**
2. Functional difficulty		1	.124**	.402**	.392**
3. Gender [ref – men]			1	0.056	.184**
4. Chronic disease status [ref – none]				1	.423**
5. Age [yrs]					1
Sample 2 [Higher income; *n* = 510]					
1. Frailty	1	.535**	.103*	.436**	.409**
2. Functional difficulty		1	-0.038	.579**	.592**
3. Gender [ref – men]			1	0.012	-0.05
4. Chronic disease status [ref – none]				1	.560**
5. Age [yrs]					1

^**^p < 0.001; *p < 0.05

Table 4 shows findings from the HLR analysis. In the baseline model (Model 1) of both samples, there was a positive association of frailty with functional difficulty (*p* < 0.001), though this association was stronger in the high-income group (β = 0.54; t = 14.28). This association is consistent between the two samples after adjusting for all the confounders in the ultimate model (i.e., model 4). Models 2 and 3 show the proportion of variance in the dependent variable explained by age and CDS, as indicated by the change in the standardized coefficient. This proportion is shown in the table as “% change in β”. Respectively, age accounted for 25% and 34% of the coefficients in samples 1 and 2, whereas CDS accounted for 28% and 35% of the coefficients in samples 1 and 2. Collectively, the two confounders accounted for 31% and 48% of the coefficients in samples 1 and 2 respectively.

**Table 4:** The associations between frailty, functional difficulty, and covariates (i.e., age and chronic disease status)

Model	Predictors	Sample 1 (Low income; *n* = 704)					Sample 2 (Higher income; *n* = 510)				
		Coefficients			95 CI	% Change in β	Coefficients			95 CI	% Change in β
		B	SE	β(t)			B	SE	β(t)		
1	(Constant)	23.59	0.77	(30.57)**	± 3.03	–-	12.67	0.75	(16.87)**	± 2.95	–-
	Frailty	1.12	0.11	0.37(10.57)**	± 0.42		1.94	0.14	0.54(14.28)**	± 0.53	
2	(Constant)	-0.38	2.79	(-0.14)	± 10.95		-13.93	2.23	(-6.25)**	± 8.76	
	Frailty	0.86	0.12	0.28(7.53)**	± 0.45	-25%	1.28	0.13	0.35(9.78)**	± 0.51	-34%
	Age (yrs)	0.40	0.05	0.32(8.71)**	± 0.18		0.52	0.04	0.45(12.48)**	± 0.16	
3	(Constant)	19.64	0.86	(22.93)**	± 3.36		12.74	0.67	(19.02)**	± 2.63	
	Frailty	0.81	0.11	0.27(7.56)**	± 0.42	-28%	1.27	0.14	0.35(9.40)**	± 0.53	-35%
	CDS (ref – none)	8.05	0.91	0.31(8.88)**	± 3.56		8.95	0.78	0.43(11.48)**	± 3.06	
4	(Constant)	-0.94	3.14	(-0.30)	± 12.33		8.08	0.81	(10.02)**	± 3.17	
	Frailty	0.82	0.12	0.26(6.58)**	± 0.49	-31%	1.01	0.13	0.28(7.84)**	± 0.50	-48%
	Age (yrs)	0.34	0.05	0.28(6.91)**	± 0.19		4.27	0.51	0.31(8.33)**	± 2.02	
	CDS (ref – none)	5.20	1.05	0.21(4.97)**	± 4.11		5.93	0.8	0.28(7.42)**	± 3.14	

^**^p < 0.001; *p < 0.05; B – unstandardised coefficient; β – standardised coefficient; *SE *standard error (of B); *CI *confidence interval (of B); *CDS *chronic disease status; “% change in β” is the percentage change in the β coefficient between frailty and functional difficulty (in model 1) due to the covariate(s) in models 2, 3, and 4; tolerance ≥ 0.6 for all predictors; total variance explained ranges from 13.6%-28.9% for sample 1 and from 28.5%-51.1% for sample 2; the F-test was significant at *p* < 0.001 for all models (blocks)

Table 5 shows the associations of frailty with functional difficulty between men and women for samples 1 and 2. In the baseline model (i.e., model 1), frailty was positively associated with functional difficulty in both samples (*p* < 0.001). This relationship for both samples is retained in the ultimate model (i.e., model 4); this association is stronger for men in the low-income group (β = 0.25; t = 4.83) but stronger for women in the high-income group (β = 0.39; t = 8.42). In models 2–4, the association between frailty and functional difficulty weakened significantly in both samples for men and women after controlling for age and CDS. Generally, the effect sizes in the higher income sample are larger, which suggests that the relationships confirmed are more likely among older adults with higher income. Thus, functional difficulty is more likely to be the consequence of frailty in the higher income sample.

**Table 5:** The association of frailty with functional difficulty between men and women

Model	Predictor	Sample 1 (Low income, *n* = 704)		Sample 2 (Higher income, *n* = 510)	
		Men	Women	Men	Women
		β(t)	β(t)	β(t)	β(t)
1	(Constant)	(20.39)**	(0.36)	(11.23)**	(12.76)**
	Frailty	0.37(7.67)**	0.36(6.98)**	0.45(7.83)**	0.63(13.22)**
2	(Constant)	(0.30)	(-0.61)	(-4.50)**	(-4.04)**
	Frailty	0.29(5.87)**	0.26(4.55)**	0.26(4.97)**	0.46(9.58)**
	Age (yrs)	0.31(6.25)**	0.33(5.83)**	0.49(9.29)**	0.39(8.05)**
3	(Constant)	(15.86)**	(16.67)**	(13.43)**	(13.86)**
	Frailty	0.29(5.84)**	0.23(4.53)**	0.21(3.60)**	0.49(10.89)**
	CDS (ref – none)	0.26(5.32)**	0.38(7.43)**	0.50(8.64)**	0.37(7.98)**
4	(Constant)	(0.91)	(0.56)	(-1.24)	(-2.56)*
	Frailty	0.25(4.83)**	0.20(3.73)**	0.19(3.41)**	0.39(8.42)**
	Age (yrs)	0.25(4.74)**	0.22(3.77)**	0.33(5.13)**	0.29(6.16)**
	CDS (ref – none)	0.17(3.13)**	0.27(4.48)**	0.28(4.10)**	0.28(6.07)**

^**^p < 0.001; *p < 0.05; β – standardised coefficient; *CDS *chronic disease status, *ref *reference; tolerance ≥ 0.7 for all predictors; total variance explained ranges from 19.8%-57.3% for sample 1 and from 28.6%-49.9% for sample 2; the F-test was significant at *p* < 0.001 for all models (blocks)

## Discussion

This study aimed to assess the association of frailty with functional difficulty between men and women with different income levels while adjusting for age and CDS.

### Main findings

Frailty was positively associated with functional difficulty, which suggests that higher frailty was associated with higher functional difficulty. This result supports the Disengagement Theory of Ageing (DTA), which posits that the individual experiences a gradual decline in functional ability due to the onset and progression of frailty in the ageing process. The theory adds that older adults with frailty are less likely to perform ADL and IADL, which implies that frailty can be associated with higher functional difficulty in old age. At odds with the DTA are the Activity Theory of Ageing (ATA) and Continuity Theory of Ageing (CTA), both of which argue that individuals can maintain social activities, functional capacity, and well-being in later life by adapting previous life experiences [32, 33]. They imply that functional ability can be maintained, and the onset of frailty delayed in later life. A review of the above theories suggests that DTA, compared to the ATA and CTA, is more valid in an African context owing to people in this context having a lower socio-economic status characterised by poverty and poor neighbourhoods.

Beyond its congruence with the DTA, our result is analogous to a positive association between frailty and functional limitation, low functional ability, or functional disability, which has been confirmed in previous research [6, 11, 12, 34]. Specifically, a cross-sectional study in Spain found that frailty in older adults was associated with low functional ability [12]. Similar findings were reached with the cross-sectional design in China [34], the Netherlands [11], and Vietnam [6]. These studies, nevertheless, were conducted in non-African contexts. Although there are related studies undertaken in Africa, no study has examined the association of frailty with functional difficult between groups with different income levels and between men and women. Thus, this study builds upon the consistency of the evidence across contexts.

Unique to this study is our confirmation of the association between frailty and functional difficulty within both samples after controlling for the two ultimate confounders. Though the relationship is consistent between the two samples, it is stronger in the higher-income sample. This outcome is noteworthy given that the higher-income sample reported lower frailty, compared with the low-income sample (see Table 1). Similarly, lower frailty reported in the higher-income sample by women was more strongly associated with functional difficulty, compared to higher frailty reported by the same group in the low-income sample. So, though women reported lower frailty compared with men in the higher-income sample, their frailty more strongly predicted functional difficulty.

The foregoing evidence can be explained from two viewpoints. Firstly, women in the higher-income group may have unique circumstances by which frailty more strongly predicts functional difficulty. For instance, working women with a higher-income status might have performed higher occupational sitting, compared with their counterpart men [35–37]. Both frailty and functional difficulty are associated with physical inactivity or a lack of exercise [18, 20, 21]. Thus, based on the literature, lower physical activity in women may have explained our result. Secondly, we possibly did not adjust for all key covariates [e.g., physical activity, sedentary behaviour, or sitting time] that may be more prevalent amongst high-income women. Future research adjusting for these potential covariates may clarify our results.

### Implications for practice

Our findings have important implications for healthcare planning, ageing, and public health policy. The provision of health services ought to be cognisant of differences in frailty amongst groups. Groups with lower frailty are not necessarily less susceptible to functional difficulty. If so, it may be misleading to assume that groups with higher frailty need to be prioritised in healthcare. Our study, thus, reveals a need for healthcare planners to assess patient needs based on differences in frailty amongst groups, and the association of frailty with functional difficulty or other risks. Our evidence also suggests that the burden of healthcare due to frailty and functional difficulty may be different for men and women. Similarly, frailty and functional difficulty may be different between men and women because of differences in access to healthcare explained by income inequalities between the two sexes.

Our sensitivity analysis implies that the association between frailty and functional difficulty is not entirely due to age and CDS; frailty may depend on other factors [e.g., early or childhood disability]. This reasoning is corroborated by studies [38, 39] that have reported and explained the prevalence of frailty in younger populations, including children. This being so, public health interventions against frailty should not be focused on older adults only, and healthcare expenditure against frailty and its associated risks should not undermine other segments of the population. As the sensitivity analysis suggests, though, age and CDS have the largest influence on the primary relationship, which means that age and CDS, compared to the other confounders, more strongly determine whether frailty would predict functional difficulty.

Healthcare organizations may need to prepare for a higher burden of care related to frailty from older adults with higher income since frailty is more likely to predict functional difficulty in this sample. Even so, future studies are needed to better understand why frailty may more strongly predict functional difficulty in older adults with higher income. Government and healthcare organizations should prepare to provide more health and social support for women on high income since frailty is more likely to be associated with functional difficulty and its health problems in this group. Similarly, they should prepare to provide more health and social support for men living on lower income, given that functional limitations and their health problems are more likely to be predicted by frailty among them.

### Limitations of the study

This study has some limitations that future researchers and decision-makers should consider. Firstly, this study as a cross-sectional design provides only associations and does not establish causality between frailty and functional difficulty. This study does not necessarily eliminate confounding bias since it could not have adjusted for all potential confounders. It is, therefore, likely that the regression weights reported in this study are confounded. Compared to samples used in some studies [20, 21], this study’s samples were relatively small. There was no relevant sampling frame for determining a representative sample, so we employed a non-probabilistic sampling method. Hence, our results may not be generalizable. Future replications of our study intended to enhance the generalisability of our findings are, therefore, warranted.

The two samples used were unequal in size, which might have resulted in higher regression coefficients and their statistical significance in the larger sample. Most related previous studies [18–21], though, produced useful findings with unequal samples, and removing some observations or participants from the larger sample to achieve equality in the samples would have resulted in bias. Experimental designs that generate and use equal samples through stratified randomisation, for example, can overcome this limitation.

### Strengths of the study

Despite the above limitations, this study has several strengths. Firstly, it is the first to compare the associations of frailty with functional difficulty between men and women over two samples with different income levels. Thus, this study improves stakeholders’ understanding of the possibility of frailty predicting functional difficulty differently in men and women and among groups with different income levels. This understanding may encourage the implementation of measures to prevent functional limitations by eliminating inequities in the income of men and women. It may also help in determining the unique healthcare needs of men and women that are the result of frailty-driven functional limitations.

This study adopted an innovative statistical strategy to systematically adjust for key personal factors, allowing researchers and stakeholders to assess the relative influences these factors may have on frailty and its association with functional difficulty. This technique can be applied to future cross-sectional analyses and is more informative than traditional methods incorporating all potential confounders at a time. Finally, our cross-sectional design follows the STROBE checklist and includes measures against CMB and confounding. The relative robustness of our cross-sectional design could, thus, be a model for future research. Appendix 3 is the STROBE checklist followed.

## Conclusion

Frailty was more strongly associated with functional difficulty in women, compared with men. Women in the low-income sample reported higher frailty, but their frailty was less strongly associated with functional difficulty. Our evidence suggests that the burden of healthcare due to frailty and functional difficulty may be different for men and women. Healthcare organizations may need to prepare for a higher burden of care related to frailty from older adults with higher income. These organizations should prepare to provide more health and social support for women on high income and men on low income.

## Supplementary Information

Supplementary Material 1.

Supplementary Material 2.

Supplementary Material 3.

Supplementary Material 4.

Supplementary Material 5.

Supplementary Material 6.

Supplementary Material 7.

## Data Availability

Data used for this study are available as an online supplementary material (i.e., supplementary materials 6 and 7).
