# Supplementary material for: Association of frailty with functional difficulty in older Ghanaians: stability between women and men in two samples with different income levels
Source: BMC Geriatr. 2024 Nov 15;24:952. doi: 10.1186/s12877-024-05534-9 (PMC11566837; doi:10.1186/s12877-024-05534-9)
Supplement: Supplementary file 1 — Supplementary Material 1. [file 12877_2024_5534_MOESM1_ESM.docx]

Appendix 1a. Items of the scale used to measure frailty

| No. | Item | Response | |
| --- | --- | --- | --- |
|  |  | No | Yes |
| 1 | Do you feel physically healthy? |  |  |
| 2 | Have you lost a lot of weight recently without wishing to do so? |  |  |
| 3 | Do you experience problems in your daily life due to difficulty in walking? |  |  |
| 4 | Do you experience problems in your daily life due to difficulty maintaining your balance? |  |  |
| 5 | Do you experience problems in your daily life due to poor hearing? |  |  |
| 6 | Do you experience problems in your daily life due to poor vision? |  |  |
| 7 | Do you experience problems in your daily life due to lack of strength in your hands? |  |  |
| 8 | Do you experience problems in your daily life due to physical tiredness? |  |  |
| 9 | Do you have problems with your memory? |  |  |
| 10 | Have you felt down during the last month? |  |  |
| 11 | Have you felt nervous or anxious during the last month? |  |  |
| 12 | Are you able to cope with problems well? |  |  |
| 13 | Do you live alone? |  |  |
| 14 | Do you sometimes miss having people around you? |  |  |
| 15 | Do you receive enough support from other people? |  |  |

**Coding**: No – 0; Yes – 1; 5 is the base cut-off score of frailty on the scale

Source: Dong et al. (2017)
