# Supplementary material for: Association of frailty with functional difficulty in older Ghanaians: stability between women and men in two samples with different income levels
Source: BMC Geriatr. 2024 Nov 15;24:952. doi: 10.1186/s12877-024-05534-9 (PMC11566837; doi:10.1186/s12877-024-05534-9)
Supplement: Supplementary file 2 — Supplementary Material 2. [file 12877_2024_5534_MOESM2_ESM.docx]

Appendix 1b. Items of the scale used to measure functional difficulty

| No. | Item | Response | | | |
| --- | --- | --- | --- | --- | --- |
|  |  | No difficulty | Somewhat difficult | Most difficult | Unable to perform |
| 1 | Lifting weight (up to 1–2 kg) |  |  |  |  |
| 2 | Bending |  |  |  |  |
| 3 | Squatting |  |  |  |  |
| 4 | Walking (up to 1–2 km) |  |  |  |  |
| 5 | Climbing |  |  |  |  |
| 6 | Arising from bed/chair |  |  |  |  |
| 7 | Household work |  |  |  |  |
| 8 | Outside work |  |  |  |  |
| 9 | Use of public transport |  |  |  |  |
| 10 | Social gathering |  |  |  |  |
| 11 | Self-cleaning (Bathing) |  |  |  |  |
| 12 | Toilet use |  |  |  |  |
| 13 | Dressing |  |  |  |  |
| 14 | Eating food |  |  |  |  |

**Note**: The scale measures the extent to which individuals found it difficult to perform self-care activities in the last week on a 4-point Likert scale. Coding: No difficulty – 1; Somewhat difficult – 2; Most difficult – 3, and Unable to perform – 4.

Source: Nagarkar et al. (2014)
