# Supplementary material for: Association of frailty with functional difficulty in older Ghanaians: stability between women and men in two samples with different income levels
Source: BMC Geriatr. 2024 Nov 15;24:952. doi: 10.1186/s12877-024-05534-9 (PMC11566837; doi:10.1186/s12877-024-05534-9)
Supplement: Supplementary file 3 — Supplementary Material 3. [file 12877_2024_5534_MOESM3_ESM.docx]

Appendix 2a. Assumptions assessed for hierarchical linear regression and steps followed to meet them

| # | Assumption | Step | Result | Decision |
| --- | --- | --- | --- | --- |
| 1 | Normality of the data associated with the dependent variable | Assessed normality with the Shapiro-Wilk’s test of normality | The Shapiro-Wilk’s test produced a non-significant result at p >0.05 (Asiamah et al., 2022). | Normality was established |
| 2 | Linearity of the associations | We plotted standardized residuals against standardized predicted values of the dependent variable in HLR analysis models fitted. | The graph shows a linear cluster of values and a straight line as recommended (Asiamah et al., 2022) | Assumption or condition was met |
| 3 | Independence of regression errors | Durbin Watson statistics were generated for all the HLR models fitted. | Durbin-Watson statistic was approximately 2 as recommended (Asiamah et al., 2022) | The assumption was met |
| 4 | Multi-collinearity | Tolerance values were computed through the HLR models. | The tolerance values are >0.2 as recommended (Asiamah et al., 2022) | The assumption was met |
| 5 | Homogeneity of variances | We plotted standardized residuals against standardized predicted values of the dependent variable in the HLR models fitted. | The graphs produced a satisfactory pattern as recommended (Asiamah et al., 2022) | The assumption was met |

**Note**: HLR – hierarchical linear regression
