# Supplementary material for: Association of frailty with functional difficulty in older Ghanaians: stability between women and men in two samples with different income levels
Source: BMC Geriatr. 2024 Nov 15;24:952. doi: 10.1186/s12877-024-05534-9 (PMC11566837; doi:10.1186/s12877-024-05534-9)
Supplement: Supplementary file 4 — Supplementary Material 4. [file 12877_2024_5534_MOESM4_ESM.docx]

Appendix 2b. Steps followed in the first sensitivity analyses for confounding variables

| Stage | # | Action |
| --- | --- | --- |
| 1 | 1 | Fit a simple linear regression model to assess the relationship between frailty and functional difficulty |
|  | 2 | Note the standardised regression weight from step 1 |
|  | 3 | Fit a multiple linear regression model in which all measured confounding variables are treated as predictors of the main independent variable, frailty |
|  | 4 | Identify from step 3 potential confounders that have a p-value ≥0.25 |
|  | 5 | Predictors from step 4 that produced a p≥0.25 should be removed from the analysis and the others kept for the next stage of the analysis |
| 2 | 6 | Adjust for each of the remaining confounding variables in the model fitted at step 1 |
|  | 7 | Compute the per cent (%) change between the standardised regression weight at step 1 and the new weight resulting from step 6 |
|  | 8 | All potential confounders that produce a change of 10% or more should be incorporated into the final analysis as the ultimate confounders |
